# Safety and effectiveness of endovascular treatment of splenic artery aneurysms: a single centre experience

**DOI:** 10.1007/s00423-025-03791-9

**Published:** 2025-07-01

**Authors:** Laura Lech, Annette Thurner, Dominik Peter, Christoph-Thomas Germer, Sven Flemming, Ralph Kickuth

**Affiliations:** 1 Department of General, Visceral, Transplant, Vascular and Paediatric Surgery, University Hospital Würzburg, Oberdürrbacherstrasse 6, 97080 Würzburg, Germany; 2 Institute of Diagnostic and Interventional Radiology, University Hospital Würzburg, Würzburg, Germany

**Keywords:** Splenic artery, Aneurysm, Endovascular treatment, Covered stent, Coil embolization

## Abstract

**Purpose:**

The aim of the study was to analyse the safety and effectiveness of endovascular treatment of splenic artery aneurysms (SAAs), and thereby complement the limited number of publications on this topic in the current literature.

**Methods:**

Twenty-three patients who underwent endovascular treatment for true splenic aneurysm or pseudoaneurysm at our centre between March 2010 and December 2024 were retrospectively included in the study. Data collection and analysis included technical and clinical success, adverse events, as well as mortality and follow up data.

**Results:**

21 true SAAs and two pseudoaneurysms with a mean diameter of 2.56 ± 0.81 cm (range, 2.0–4.0 cm) were included in the study. One SAA was treated with covered stent implantation, nine with sac embolization, and 13 with intra-aneurysm and aneurysm-bearing artery coil embolization. The technical success rate was 100%. The clinical success rate was 91.30%, defined as persistent aneurysm exclusion and preservation of at least 50% splenic perfusion. Mild adverse events occurred in 34.78% of patients, including post-embolization syndrome (21.74%) and small splenic infarctions (13.04%). One patient experienced post-embolization pancreatitis (4.35%). Severe adverse events (8.70%) included two splenectomies due to a splenic abscess and gastric bleeding caused by coil migration. No deaths occurred during the study period.

**Conclusion:**

Endovascular treatment is an effective, minimally invasive alternative to open surgery for splenic artery aneurysms, achieving high success rates and satisfactory follow-up outcomes when the appropriate method is chosen—both for asymptomatic SAAs and hemodynamically stable patients with ruptured SAAs or pseudoaneurysms.

## Introduction

Splenic artery aneurysms are rare vascular anomalies and the most common type of visceral aneurysm [1–10]. Most SAAs are asymptomatic and are increasingly detected incidentally with the use of advanced imaging techniques such as computed tomography angiography (CTA) or ultrasound [11]. The risk of complications, especially ruptures, increases with aneurysm size. For aneurysms larger than 2 cm, the risk of ruptures increases, especially if there is an aneurysm growth [1–9]. A ruptured SAA carries a high mortality rate of 10–25% in non-pregnant patients und up to 70% during pregnancy [1, 6, 10]. This is primarily due to the life-threatening haemorrhage, which occurs in approximately 10% of cases [1]. Therefore, there is a common consensus on the treatment indications for SAAs, which include: (a) a diameter of a true SAA ≥ 3 cm; (b) a size increase of 0.5 cm per year; (c) symptomatic SAA regardless of size; (d) ruptured aneurysm or (e) pseudoaneurysm [1, 3, 6–8, 10, 12]. Additionally, a SAA should be treated in women of childbearing age and during pregnancy, as well as in patients with portal hypertension regardless of size [6, 10].

Traditionally, SAAs have been managed with open surgery [1–10]. However, endovascular therapies, such as coil embolization and covered stent implantation, have emerged as minimally invasive alternatives in recent years [1, 5–10]. The current literature includes few large retrospective studies that analyse different endovascular techniques for SAA management and present long-term outcomes [1, 5, 6, 8, 9]. This study aims to address this gap and enhance the understanding of endovascular SAA treatment.

## Materials and methods

### Study designed patients and aneurysm characteristics

This retrospective, single-centre study was conducted at the departments of interventional radiology and vascular surgery at the University Hospital of Wuerzburg between March 2010 and December 2024 and included all patients who underwent endovascular treatment for SAAs. The inclusion criteria were as follows: (a) age over 18 years; (b) the ability to provide informed consent; (c) diameter of a SAA ≥ 2 cm; (d) increase of diameter of more than 0.5 cm/year; (e) symptomatic SAA regardless of the diameter; and (f) ruptured aneurysm or (g) pseudoaneurysm. Patients who previously underwent open surgery for a splenic artery aneurysm or those who were unable to provide informed consent were excluded from the study. Data on demographics, comorbid conditions and the patients’ clinical and imaging follow-up were retrospectively analysed within the clinical information system and the picture archive and communication system.

**Fig. 1:**
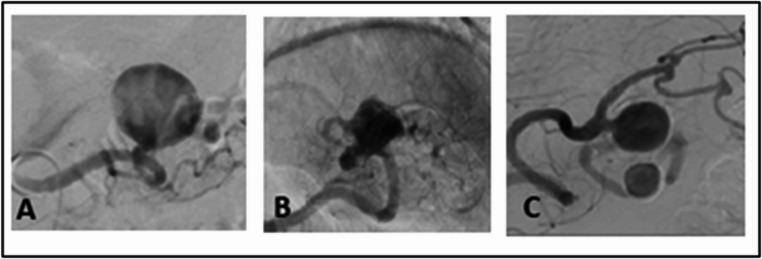
Some SAA Types following the classification by Shu et al. (**A**) Type I with a narrow neck; (**B**) Type III in the region of the splenic hilum; (**C**) Type IVc tandem aneurysms

The SAAs were further categorized into five subtypes based on their radiological morphology, following the classification by Shu et al. [8]. In this system, type I aneurysms are found within the main splenic artery branch with a narrow neck (≤ 0.5 cm). Type II aneurysms, also located in the main branch segment, have a wide neck (> 0.5 cm). Type III aneurysms occur within the secondary branches or at the splenic hilum. Type IV aneurysms are divided into three subgroups: type IVa are located at the splenic artery ostium or a deviated splenic artery from the superior mesenteric artery; type IVb refers to giant aneurysms with a diameter greater than 5 cm, and type IVc includes tandem aneurysms or pronounced tortuosity of the splenic artery (Fig. 1). Type V aneurysms are classified as pseudoaneurysms or ruptured SAAs.

### Ethics approval

Ethical approval was given by the Ethics Committee of the University of Würzburg.

### Intervention

All procedures were performed by one experienced interventional radiologist with over 28 years of experience in embolization. Under local anaesthesia, digital subtraction angiography was conducted via a common femoral artery approach by using a 5-F vascular sheath (Terumo, Tokyo, Japan). All embolizations were carried out in a dedicated angiography suite (AXIOM Artis zee, SIEMENS Healthcare, Forchheim, Germany or Azurion Clarity IQ, Philips Medical Systems, Best, The Netherlands). A Cobra- or Sidewinder-shaped catheter (4 − 5-F C-2 or VS-2 catheter; Cook Medical, Bjaeverskov, Denmark) was used for selective catheterization of celiac trunk and splenic artery, respectively. Diagnostic catheter manoeuvres were usually performed with a 0.035-inch guidewire (Radifocus; Terumo, Tokyo, Japan). Once an aneurysm, its location and morphology had been verified, the patients underwent super-selective trans-catheter arterial embolization (TAE) with a coaxially placed 2.4- or 2.7-F microcatheter (Progreat, Terumo, Tokyo, Japan) or covered stent implantation. In case of TAE simple sac embolization or intra-aneurysm and aneurysm-bearing artery coil embolization with occlusion of the inflow and outflow tracts of the aneurysm was conducted. For coil embolization, both pushable coils, e.g. Tornado or Nester (both Cook Medical, Bjaeverskov, Denmark), and detachable coils e.g. Ruby (Penumbra, Alameda, CA, USA) or Concerto (Medtronic, Minneapolis, MN, USA) were utilized, with eight patients receiving a combination of both types. Covered stent placement was performed exclusively using the Advanta V12 stent (Atrium Medical, Hudson, NH, USA).

## Follow-up

Post-interventional, all patients remained hospitalized for a minimum of 24 h for clinical monitoring. After discharge, patients were followed at 6- and 12-months post-intervention, with annual check-ups thereafter. Follow-up assessments included clinical evaluations and imaging studies, primarily duplex ultrasound and CTA. Further clinical and imaging follow-up was available in 10 of the 23 patients (43.48%). In these patients, the mean follow-up time was 50.4 months (range, 18–108 months). Patients were advised to immediately contact the outpatient clinic at the onset of new or recurrent clinical symptoms.

### Definition of study outcomes

**Fig. 2:**
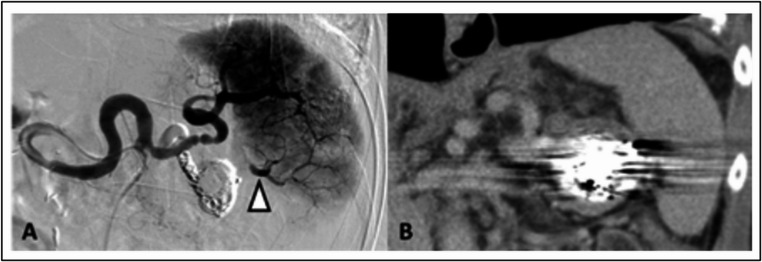
Technical success after coil embolization of the SAA. (**A**) Digital subtraction angiography confirming complete exclusion of the sac and the aneurysm-bearing main inferior branch artery with preserved perfusion of the spleen; note retrograde perfusion of the inferior lobar branches via anastomosing intrasplenic segmental branches from the main superior branch artery (white arrowhead). (**B**) Coronal contrast enhanced computed tomography image in the venous phase four days after SSA embolization confirming preserved perfusion of the entire spleen

Technical success was defined as the absence of perfusion in the SAA with preserved splenic perfusion, as observed in the final angiography (Fig. 2). Clinical success was determined by the sustained exclusion of the aneurysm and the preservation of at least 50% of splenic perfusion through collaterals or the main branch of the splenic artery, as seen in ultrasound or CTA during follow-up examinations. Complications, re-interventions, and mortality were documented and analysed. Adverse events (AEs) were categorized according to the reporting standards of the Society of Interventional Radiology (SIR) [13].

### Statistical analysis

The results of this study were presented descriptively. Categorical variables are expressed as counts and percentages. Continuous variables are reported as mean ± standard deviation or as median and interquartile range, depending on the normality of the distribution. Due to the small sample sizes, no comparative tests were performed, as the study lacked sufficient power for reliable statistical analysis.

## Results

### Demographic data and comorbid conditions

23 patients were included in the present study. The mean age was 63.0 ± 12.41 years (range, 31–81 years). Among these patients, 10 were women (43.48%) and 13 were men (56.52%). Three patients (13.04%) had additional aneurysms in other vascular territories; one in the common iliac artery and two in the infrarenal aorta. Comorbidities are presented in Table 1. The most common comorbidity was arterial hypertension, present in 13 patients (56.52%). Five patients (21.74%) had a smoking history.

**Table 1:** Baseline characteristics of patients and splenic artery aneurysms - Values are reported as n (%), median (range)

Demographics	Number
**Gender**	
Male	13 (56.52)
Female	10 (43.48)
**Age** (years; median, range)	63.0 (31–81)
**Comorbidities**	
Hypertension	13 (56.52)
Diabetes mellitus	3 (13.04)
Coronary heart disease	3 (13.04)
Chronic pancreatitis	1 (4.35)
Intraductal papillary mucinous neoplasm	1 (4.35)
Chronic obstructive pulmonary disease	3 (13.04)
Dialysis	1 (4.35)
Other aneurysms (aortic, iliac aneurysm)	3 (13.04)
Tabaco use	5 (21.74)
**Symptoms**	
Symptomatic	4 (17.39)
Asymptomatic	19 (82.61)
Rupture	3 (13.04)
**Type of SAAs**	
True	21 (91.30)
Pseudoaneurysma	2 (8,70)
**Classification of SAAs**	
Type I	3 (13.04)
Type II	7 (30.34)
Type III	3 (13.04)
Type IVa	1 (4.35)
Type IVb	0
Type IVc	4 (17.39)
Type V	5 (21.74)
**Aneurysm neck**	
narrow	9 (39.13)
wide	14 (60.87)

### Aneurysm-specific characteristics

At the time of intervention, the mean maximum aneurysm diameter was 2.56 ± 0.81 cm (range, 2.0–4.0 cm). Four of the 23 patients (17.39%) were symptomatic and three of these four had a rupture. The aneurysms were categorized into true aneurysms (*n* = 21, 91.30%) and pseudoaneurysms (*n* = 2, 8.70%). One pseudoaneurysm resulted from acute on chronic pancreatitis and an intraductal papillary mucinous neoplasm (IPMN), while the second resulted from a traumatic splenic laceration. Radiologically, 14 (60.87%) of the SAAs had a wide-neck aneurysm, while nine (39.13%) were classified as narrow-neck aneurysms. Furthermore, the aneurysms were categorized based on their morphology into five subtypes, following the classification published by Shu et al. [8] (Table 1).

### Treatment and hospitalisation

Of the 23 included patients, 22 aneurysms were successfully excluded using coil embolization, whereas a single aneurysm (Type IVa) was treated with covered stent implantation. Nine patients (39.13%) underwent isolated sac embolization of their aneurysmal sac. 13 patients (56.52%) underwent intra-aneurysm and aneurysm-bearing artery coil embolization. A median of 17 coils (range, 6–38 coils) were utilized for the exclusion of a SAA. Seven (31.82%) SAAs were embolized using pushable coils and seven further SAAs (31.82%) using detachable coils. In the remaining eight cases (36.3%), a combination of both pushable and detachable coils was employed. Complications during the interventions did not occur. No intervention related SAA ruptures were observed during hospitalisation. Conversion to open surgery was not necessary. The median length of hospital stay was 3 days (range, 1–10 days) (Table 2).

**Table 2:** Embolization technique and study Endpoints - Values are reported as n (%), median (range)

Variables	Number
**Embolization technique** Coil embolization - sac embolization - intra-aneurysm and aneurysm-bearing artery coil embolization Stent Implantation	23 (100) 22 (95.65) 9 (39.13) 13 (56.52) 1 (4.35)
**Technical success**	23 (100)
**Clinical success**	21 (91.30)
**Mild adverse events** Postembolization syndrome Small splenic infarct	8 (34.78) 5 (62.50) 3 (37.50)
**Moderate adverse events** Pancreatitis	1 (4.35) 1 (4.35)
**Severe adverse events** Splenic abscess Gastric bleeding	2 (8.70) 1 (50.00) 1 (50.00)
**Splenectomy**	2 (8.70)
**Mortality**	0
**Re-intervention**	0
**Hospital Stay** (days, median (range))	3 (1–10)

### Study outcomes

The final angiography showed a sufficiently eliminated SAA with preserved perfusion of the spleen in all patients (*n* = 23), resulting in a technical success rate of 100% (Table 2). Regular radiological follow-up revealed persistent exclusion of the aneurysms and preservation of perfusion of at least 50% of the spleen in 21 patients, resulting in a clinical success rate of 91.30%. Two patients (8.70%) experienced clinical failure due to splenectomy, which became necessary because of adverse events. Re-intervention due to aneurysm recanalization was not necessary. There were no complications in the inguinal access area, such as secondary haemorrhages, inguinal haematomas or pseudoaneurysms of the common femoral artery.

Eight AEs (34.78%) were identified during the follow-up period. Five of these eight patients (62.50%) reported abdominal pain due to post-embolization syndrome during their hospitalization. In three patients (37.50%) — one with a type II, one with a type III, and one with a type IVc SAA — ultrasound follow-up revealed small splenic infarctions, without clinical symptoms or need of treatment. Therefore, these splenic infarctions are classified as mild AE. Embolization of a type III SAA resulted in post-interventional pancreatitis, requiring intensified analgesia and fluid substitution, classified as a moderate AE (4.35%). Two patients suffered from severe AE (8,70%). One patient developed an extended (9 × 8 × 8 cm) splenic abscess with reactive pleural effusion and pleuritis after embolization of his type II SAA. The second severe AE was an upper gastrointestinal haemorrhage. It occurred in the patient with acute on chronic pancreatitis and IPMN. The pancreatitis in this patient led to both an erosion-related pseudoaneurysm of the splenic artery and an erosion-related lesion of the stomach. After embolization of the pseudoaneurysm, coil migration occurred in the gastric lesion and thus to gastric haemorrhage, requiring emergency surgery, including simultaneous splenectomy, gastrectomy and pancreaticoduodenectomy. In both cases of severe AEs, a splenectomy was necessary. No patients died during the study period (Table 2).

## Discussion

In this study, the technical success rate was 100%, with final angiography in 23 patients showing sufficient exclusion of the SAA and preserved splenic perfusion (Fig. 2). These findings are in line with the current literature, where endovascular SAA treatment success rates range from 90 to 100% [1, 5, 6, 8, 9]. The study also demonstrated a high clinical success rate of 91.30% (*n* = 21), with sufficient SAA exclusion and at least 50% spleen perfusion preserved at follow-up. Two patients (8.70%) experienced a clinical failure, as a splenectomy was necessary postinterventionally. A direct comparison with current literature is challenging, as most studies do not clearly define or report clinical success. However, regarding re-intervention rates, the literature reports some aneurysms that showed reperfusion at follow-up, mainly via collateral vessels of the short gastric arteries [5, 9]. In the literature, the re-intervention rates of visceral artery aneurysms treated endovascular are generally low ranging from 6.3 to 26% [14]. Like in other studies, re-interventions had not to be performed in the present study [6, 8, 9]. The large meta-analysis (> 1.300 included patients) by Hogendoorn et al. compared open surgical treatment with endovascular therapy for SAA [5]. Although, this meta-analysis showed high technical success rates for both procedures (open group: 97.8% and endovascular group: 95.2%), it revealed a higher re-intervention rate in the endovascular group compared to the open group (3.2% per year vs. 0.5% per year) [5]. Most patients in the open group underwent aneurysm resection with simultaneous splenectomy or reconstruction of the splenic artery without splenectomy [5]. In open procedures, the aneurysm is resected, which prevents any further reperfusion during follow-up. This could explain the low re-intervention rates. In contrast, after embolization, the SAA remain, and reperfusion can occur over time, often requiring additional re-interventions.

Thus, open surgery is associated with decreased re-intervention rate in the follow up, but endovascular treatment of SAAs is accompanied by better short-term results [5]. A major advantage of the endovascular intervention is the minimally invasive therapy, resulting in significantly shorter hospital stays. The median hospital stay ranged from 2.0 to 5.0 days, which is notably shorter than that for open surgical treatment (mean 9.8 days) [1, 3, 5, 6, 8, 9], aligning with the present results (median 3, range 1–10 days). In the current study, mild AEs occurred most frequently (34.78%), without causing significant harm to the patients. The post-embolization syndrome was the most common complication in this study (21.74%). This finding is in line with the rates of PES after embolization of a SAA described in the literature, ranging from 25 to 30% [1, 5, 6, 8, 9]. PES is characterized by post-interventional abdominal pain, with or without fever, and usually resolves with symptom-targeted analgesia [1, 5, 6, 9]. A small splenic infarction was observed in a few study patients (13.04%) as a secondary mild AE. In the literature, small splenic infarcts are frequently reported, with prevalence rates ranging from 11 to 25% [1, 6, 9]. Due to adequate arterial collateral circulation, these infarcts typically do not result in significant therapeutic consequences.

In contrast, splenic abscesses, which often arise from larger splenic infarctions, are considered more serious and generally require longer and more intensive treatments, and thus, splenic abscesses are classified as severe AEs. According to current literature, the occurrence of splenic abscesses is relatively rare [1, 6, 9]. In the present study, one patient developed a relatively large (9.0 × 8.0 × 8.0 cm) splenic abscess. Initial management involved targeted antibiotic therapy and CT-guided drainage. However, due to the progression of the condition, splenectomy ultimately became necessary. It cannot be determined retrospectively whether a superinfection caused by the CT-guided drainage in this patient aggravated the pre-existing splenic abscess and finally caused splenectomy. Three primary approaches are principally available for the treatment of splenic abscesses: conservative antibiotic therapy, minimally invasive percutaneous drainage, and splenectomy [15, 16]. Due to the low incidence of splenic abscesses (0.1–0.7%) only a few small retrospective studies have compared these methods [15, 16]. None have identified clear advantages for any particular approach. Alvi et al. demonstrated that primary splenectomy may offer superior outcomes for abscesses larger than 10 cm in diameter [15]. According to Sreekar et al., percutaneous drainage represents a safe and effective alternative to surgical intervention, particularly for unilocular abscesses, thus allowing for spleen preservation [16]. Nevertheless, there is a consensus that splenectomy is typically required in cases of large or multiple abscesses, as well as when drainage attempts fail [15, 16]. Since there are no specific guidelines regarding the treatment of splenic artery aneurysms, therapy should always be individualized based on certain factors, such as abscess size, number of abscesses, location, and septation.

Severe AEs, such as splenic abscess, are rare in endovascular SAA treatment, as seen in the present study (8.70%). Hogendoorn et al. were also unable to demonstrate any significant differences in major complications between open and endovascular approaches [5]. While a simultaneous splenectomy is often performed in open SAA repair, endovascular treatment has reduced splenectomy rates in SAA management. According to current literature, splenectomy is rarely needed after endovascular SAA treatment (< 2.0%) [1, 6, 8, 9]. This is of significant relevance, as a splenectomy is associated with increased risk of early postoperative infectious complications, particularly overwhelming post-splenectomy infection (OPSI). Although OPSI is rare, the risk of infection is over 50 times higher in post-splenectomy patients compared to the general population [17]. OPSI is a sepsis-like condition that can be life-threatening, with a mortality rate ranging from 50 to 70% without prompt intervention [18, 19]. In the present study, no OPSI was detected during follow-up. Unfortunately, no specific studies address OPSI in relation to SAA treatment. In consequence no conclusions can be drawn regarding the risk reduction of OPSI through endovascular treatment. One advantage of endovascular SAA treatment over open surgery is the significantly lower 30-day, late, and combined mortality rates [5]. Both in the present study and in many previous investigations, no deaths were observed following endovascular treatment [1, 6, 8, 9].

The Society for Vascular Surgery clinical practice guidelines on the management of visceral artery aneurysms from 2020 recommends a treatment of true asymptomatic splenic artery aneurysms at a size of > 3 cm (19). In the study presented here the mean diameter was 2.56 ± 0.81 cm, and thus, comparable with other studies evaluating endovascular approach for splenic aneurysm (1,5,9). Furthermore, it must mention that the quality of evidence for this recommendation is low and based on data published before 2020. In the meantime, the knowledge about endovascular approaches has been improved suggesting that treatment of splenic aneurysm is performed in an earlier state due to minimal-invasive technique. Thus, it remains worthy to discuss which size of diameter is the perfect threshold for conservative and interventional/operative treatment. Here further comparative studies are mandatory to draw a conclusion.

Endovascular treatment methods are typically selected based on the finding provided by imaging, particularly considering the location, size, morphology, and afferent and efferent vessels of the SAA. Many recent publications discussed which endovascular technique represents the optimal treatment for specific SAAs [1, 6, 8]. Shu et al. even developed a structured classification system for SAAs based on imaging, offering an optimized treatment strategy [8]. There is a consensus that the technical and clinical success largely depends on choosing the appropriate endovascular method for specific SAAs [1, 6, 8]. The results of the present study are comparable with those in the current literature. For saccular SAAs with a small neck (Type I), isolated sac embolization is recommended when stent implantation is not possible, due to its high technical and clinical success and low complication rate [1, 8]. On the other hand, isolated sac embolization does not seem to feasibel in for SAAs with a wide neck (Type II) or pronounced inflow and outflow tracts, as it poses a high risk of residual perfusion and coil migration [1, 8]. In such cases, intra-aneurysmal and aneurysm-bearing artery coil embolization is preferred. However, a disadvantage is the post-interventional formation of splenic infarcts of varying severity [1, 6, 8]. Despite the spleen’s preformed collateralization via short gastric or transverse pancreatic arteries, embolization of the aneurysm-bearing artery often leads to splenic infarcts [1, 6, 8]. As mentioned earlier, three small splenic infarcts and one large splenic abscess occurred after this procedure, resulting in an infarction rate of 28.57% (*n* = 4/14). Wang et al. also reported a significantly higher risk of splenic infarction following embolization of the aneurysm-bearing artery [1]. While effective, this method carries a higher complication rate than sac embolization and stent implantation [1, 8]. Its challenge lies in embolizing as much as necessary and as little as possible, requiring interventional expertise. Covered stent implantation, a low-complication method, is rarely feasible due to insufficient landing zones and tortuous splenic arteries. In this study, stent implantation was used only for one SAA in the proximal splenic artery. While we cannot provide significant results for this method, current literature recommends stent implantation as the ideal endovascular procedure when feasible, due to its safety, practicality, low complication rate, preserved perfusion and high technical and clinical success [1, 8].

Patients with pseudoaneurysms or ruptured SAAs pose significant challenges, often being multimorbid with conditions such as chronic pancreatitis, pancreatic tumour or fistula, and severe abdominal trauma. These critically ill patients are at high risk of haemorrhagic shock [1]. Therefore, treatment is time-critical for life-saving intervention [1, 8]. Per consensus it has been established that unstable patients require emergency laparotomy, while endovascular treatment is increasingly used for hemodynamically stable patients [1, 8]. In this study, five patients with type V SAAs were treated: two with pseudoaneurysms and three with ruptured SAAs. All aneurysms were successfully treated with intra-aneurysm and aneurysm-bearing artery coil embolization, achieving a technical success rate of 100%. The current literature also recommends coil embolization of both the intra-aneurysmal sac and the aneurysm-bearing artery, as isolated sac embolization in Type V SAA carries a high risk of rupture [6, 8]. In these cases, the focus is on ensuring the patient’s survival, regardless of potential post-procedural complications such as splenic infarction due to the embolization of the aneurysm-bearing artery. In our study, all five patients survived the procedure, and the clinical success was high in the follow-up (80%). Certainly, the expertise of our interventional radiologist contributed to these positive outcomes. Based on these results, endovascular treatment should be considered for hemodynamically stable Type V SAAs, if it can be promptly performed.

### Limitations

As a limitation, this study is retrospective, conducted at a single centre with a small sample size, which is reflective of the low incidence of splenic artery aneurysms (SAAs). The median follow-up period of one year is relatively short; however, a regular follow-up was successfully completed for all patients. Furthermore, due to the limited number of cases, a comparative analysis between stent and coil embolization could not be performed, as the sample size lacked sufficient statistical power for reliable conclusions.

## Conclusion

In conclusion, endovascular treatment is an effective, minimally invasive alternative to open surgery for splenic artery aneurysms (SAAs) with high technical and clinical success rates. This study is in line with the current literature, highlighting the growing use of endovascular procedures due to their favourable outcomes.

## Data Availability

No datasets were generated or analysed during the current study.
